# Anatomical relationship of pterygoid process pneumatization and vidian canal

**DOI:** 10.1016/j.bjorl.2020.06.005

**Published:** 2020-07-21

**Authors:** Nanditha Lakshman, S. Viveka, Fahad Bapu Thondupadath Assanar

**Affiliations:** a Azeezia Institute of Medical Sciences, Kollam, India; b Azeezia Institute of Medical Sciences, Department of Anatomy, Kollam, India; c Azeezia Institute of Medical Sciences, Department of Radiology, Kollam, India

**Keywords:** Vidian canal, Sphenoid bone, CT scanning, Pneumatization

## Abstract

**Introduction:**

The vidian canal acts as landmark for the identification of the petrous carotid artery, especially during extended endoscopic endonasal approaches in cranial base surgeries. In order to localize the canal and to understand the relationship of pneumatization of pterygoid process to the type of vidian canal, this study was designed.

**Objectives:**

The objective was to describe the anatomical relationship of pneumatization of the pterygoid process with types of vidian canal. The length of vidian canal, relationship to medial plate of pterygoid process and relationship to the petrous part of internal carotid artery were evaluated.

**Methods:**

Head computer tomography scans of 52 individuals for suspected paranasal pathology were studied. The degree of sphenoid sinus pneumatization, pterygoid process pneumatization and types of vidian canal (type 1, 2 and 3) were noted. The length of vidian canal, distance from the plane of medial pterygoid plate and relation of vidian canal to the junction of petrous and Gasserian (ascending) part of internal carotid artery was noted.

**Results:**

46 (92%) sphenoid sinuses were of the sellar variety. Out of 104 sides that were studied, 57 sides demonstrated a pneumatised pterygoid process and 47 were not pneumatised. In 49 sides (47.1%) the vidian canal was on the same plane as that of the medial pterygoid plate in the coronal section. The vidian canal partially protruded into the sphenoid sinus (type 2) was the most common type (50.9%), found both on right and left sides. There is a statistically significant association between the pterygoid process pneumatization and occurrence of type 2 and type 3 vidian canal configuration. The average length of the vidian canal was 16.16 ± 1.8 mm. In 96 sides, the anterior end of vidian canal was inferolateral to petrous part of internal carotid artery in the coronal plane.

**Conclusion:**

Pneumatization of the pterygoid process indicates either type 2 or type 3 vidian canal configuration.

## Introduction

The vidian canal or pterygoid canal is an osseous tunnel close to the sphenoid sinus base. The greater superficial petrosal nerve and deep petrosal nerves unite to form the vidian nerve. The greater superficial petrosal nerve conveys parasympathetic fibres from genu of the facial nerve. Deep petrosal nerve carries sympathetic fibres from the plexus around the internal carotid artery. Along with the vidian nerve, the artery of pterygoid canal (vidian artery), a branch of pterygopalatine part of maxillary artery, also passes through the Vidian canal. The bony tunnel may be a complete osseous tunnel or there may be dehiscence along its path, exposing the contents to the sphenoid sinus. The localization of the vidian canal in imaging slices during computerized tomography (CT) is of significance during evaluation of pathologic condition of paranasal sinuses.^1^, ^2^

The vidian canal is located at the junction of the pterygoid process and body of sphenoid bone.^3^ The degree of pneumatization of the pterygoid process influences the relative position of the vidian canal and may indirectly determine the type of vidian canal.^4^ The vidian canal may be completely embedded in the bony mass of sphenoid base, partly protruded from the base, or may be completely protruded in the sinus with a stalk.^5^

The vidian canal acts as landmark for the identification of petrous carotid artery especially during extended endoscopic endonasal approaches in cranial base surgeries.^6^ The extended endonasal approach is used for accessing the middle cranial fossa, mid- part of clivus, and parts of cavernous sinus.^7^ Identification of anterior genu of internal carotid artery is important during such extended approaches.^8^ The vidian canal is consistently related to the petrous part of internal carotid artery. The vidian canal indicates the point of transition from the horizontal petrous part of internal carotid artery and vertical paraclival segment at the foramen lacerum. There are many reports detailing the relative distances of the vidian canal from the nearby important land marks like the foramen rotundum, foramen lacerum, palatovaginal canal, superior and inferior sphenoid sinus walls and vomerine crest.^4^, ^5^, ^6^, ^9^

With the advent of extensive use of endoscopic sphenoid sinus surgeries from sella- related pathologies, the relevant anatomy and relations of this region have gained much research interest around the world. In this regard, a detailed evaluation of sphenoid sinus and sellar structures in every patient is pivotal in understanding the individual variations. There are many research reports in the past highlighting these details. Particularly, endoscopic vidian neurectomy – a procedure performed for vasomotor rhinitis, requires thorough pre-operative CT evaluation of patients to select the proper surgical approach.^10^ Since this method is associated with less morbidity than the traditional transantral approach to vidian canal contents, it is the favoured surgical intervention for vasomotor rhinitis where nonsurgical medical line of management has consistently failed.^11^, ^12^ Also, to resect the tumors of the pterygopalatine fossa, understanding of the vidian canal location, variations and related structures like position and location of foramen rotundum in the posterior wall of pterygopalatine fossa is crucial.^13^

Vidian canal anatomy, its relations with the pterygoid process and variations are important for all sellar related surgeries, for endoscopic vidian neurectomy procedures and pterygopalatine fossa pathologies. However, there are no major studies of such valuable anatomic data for surgeons. The vidian canal and related structures are not routinely identified and reported in the paranasal sinus CT evaluation scans unless a specific pathologic abnormality is evaluated. Therefore, in order to easily localize the canal and to understand the relationship of pneumatization of pterygoid process on the type of vidian canal, this study was designed.

Pneumatization of the pterygoid process influences the type of vidian canal. Lee et al., while studying techniques for surgical access to vidian neurectomy, have classified the vidian canal into three types.^14^ Accordingly, Type 3 has canal completely embedded within the sphenoid sinus. Type 2 is partially embedded and Type 1 has completely embedded vidian canals into the floor of sphenoid sinus. Lee at al., have found Type 2 variety as most common configuration. Yegin et al. in addition to vidian canal types have also studied the different configurations of sphenoid sinus floor.^2^ They report almost equal incidence of Type 1 and Type 3 varieties. In addition, they also note dehiscent vidian canal in nearly one fourth of all scans studied. There is wide disparity in reports on vidian canal types. Kazhayasi et al. have also noted that pterygoid process pneumatization influences the degree of protrusion of the vidian canal.^15^ In their report, they note wide variation in types of vidian canal within individual sides.

There are reports evaluating the relationship of the petrous part of internal carotid artery to vidian canal. The vidian artery may arise from the distal part of maxillary artery or from the petrous part of internal carotid artery.^16^ This artery participates in anastomoses in the pterygopalatine fossa and oropharyngeal mucosa. The vidian canal is consistently related to the second bend of the petrous part of internal carotid artery. The internal carotid artery passes through the carotid canal and into the foramen lacerum. The sympathetic fibers pass within the tunica adventitia of the artery as the deep petrosal nerve and connect to the greater petrosal nerve. The united nerve continues within a bony canal, now referred to as vidian nerve or nerve of pterygoid canal.^17^ The relationship of the vidian canal with genu of petrous internal carotid artery has been inconsistently reported. Adin et al. reports that most canals (89%) are inferior to the internal carotid artery.^18^ Whereas, Mason et al. reports 66% of the canals are inferior to the internal carotid artery.^19^ During evaluation of CT angiograms for internal carotid artery segments, Gasserian segment (the ascending segment in the cavernous sinus) is defined based on the location and relation of the vidian canal. The junction between the petrous and Gasserian (ascending) segments is marked by the posterior end of vidian canal. In a study exclusively evaluating the segments of internal carotid artery the vidian canal was found directly below this junction in 90% of the CT scans.^8^ As there are wide variations in these reports and there are no substantial data from the Indian population, this study was designed to evaluate the relationship of the vidian canal with petrous part of internal carotid artery.

With this background, this study was designed with an objective to describe the anatomical relationship of pneumatization of pterygoid process with types of vidian canal. The length of vidian canal, relationship to medial plate of pterygoid process and relation to petrous part of internal carotid artery were evaluated.

## Methods

Computer tomography scans of 52 individuals who had undergone radiological evaluation of head were studied. Institutional ethics committee approved (letter number AEC/Rev/2019/03 dated 18/01/2019) the proposal before starting the study with waiver of informed consent process. Care was taken not to reveal the patient identification data.

### Inclusion criteria

Patients who had undergone CT scan study for suspected paranasal pathology was studied in Department of Radiodiagnosis.

### Exclusion criteria

Patients who have undergone corrective surgical intervention for proven paranasal pathology were excluded.

### Images

The CT images were acquired using a high speed 160 multislice CT Aquilion Prime (Toshiba Medical Systems, India). The following study parameters were applied: exposure 120 kV, 74 mA, 60 mAs; rotation time 0.5 s; slice thickness 0.5 mm. Patient’s sex and age data were acquired from patient files.

Images were examined under three planes – coronal, sagittal and transverse. All measurements were taken using a measuring tool provided along with the machine. Measurements were in millimetre with lowest count of 0.1 mm. All bilateral measurements were performed symmetrically. Twice the same observer took each measurement; in the case of any discrepancies, the mean of the two values was recorded. All measurements were repeated in every case first by the student and next by the guide.

### Degree of sphenoid pneumatization

The sphenoid sinus was categorized into three types based on pneumatization as assessed by sagittal CT slice (Table 1).

Sellar type has well pneumatised sphenoid sinus with sellar indentation extending posteriorly into the clivus (most common variety)

Presellar type has moderate pneumatization with no sellar indentation within the sphenoid sinus.

Conchal type has no pneumatization of sphenoid sinus.

### Pterygoid process pneumatization

Presence or absence of pterygoid process pneumatization was noted. If the sphenoid sinus pneumatization extended into the pterygoid process it was considered as a pneumatised pterygoid process.

### Relation of vidian canal to medial pterygoid plate

Location of vidian canal medial, lateral or in the same plane of the medial pterygoid plate is noted. The distance (in mm) from the medial pterygoid plate was measured separately on the right side and left side.

### Vidian canal typet

Tree types of vidian canal was defined according to Lee et al.^14^

Type 1: vidian canal located within the bony floor of sphenoid sinus.

Type 2: vidian canal partially protruded into the sphenoid sinus.

Type 3: vidian canal totally protruded into the sphenoid sinus and a stalk is present.

### Length of vidian canal

The length of vidian canal was measured in axial plane from posterior wall of pterygopalatine fossa to the posterior limit of carotid canal. The length was measured along medial aspect of vidian canal.

Relationship of vidian canal with petrous part of internal carotid artery and the relationship of anterior end of vidian canal to the internal carotid artery (petrous part) in the coronal plane was noted. Expected locations were inferolateral, inferior and inferomedial.

### Sample size calculation

Pneumatization of the pterygoid process is most commonly seen with type 3 variety of vidian canal (95%) as reported in one of the recent studies.^5^ For confidence interval of 90% with precision 5%, with prevalence of 95% the sample size required is calculated as 52.

### Statistical analysis

The data was tabulated and analysed using MS Excel. All continuous data were expressed as mean with standard deviation. Comparisons were done by Student *t*-test and values < 0.05 were taken as significant. The significance of association between type of vidian canal and pterygoid process pneumatization was noted with Fisher’s exact probability test and Chi-Square test.

## Results

Fifty-two patients who had undergone CT scan study for suspected paranasal pathology were studied. The mean age was 37.44 (Standard Deviation ±13.72, maximum age – 80 years, minimum age – 15 years). Out of these patients, 30 were males and 22 were females.

**Figure 1:**
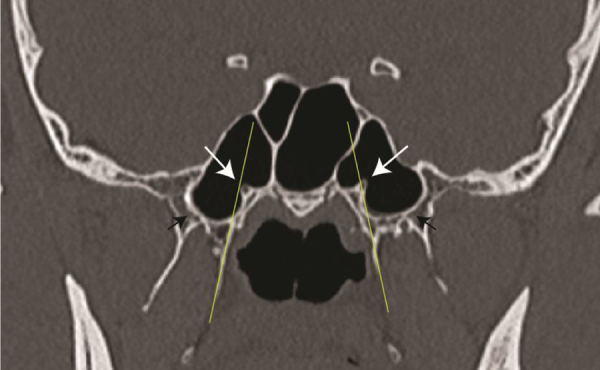
Coronal CT scan showing the position of the vidian canal (white arrow) in the same plane as that of the medial pterygoid plate (thin yellow line) on the left side and medial to this plane on the right side. Black arrows indicated a pneumatised pterygoid plate.

Forty-six (92%) sphenoid sinuses were sellar variety and 6 (12%) were presellar type. We did not find conchal variety of sphenoid sinus. Out of 104 sides that were studied, 57 sides were pneumatised pterygoid process and 47 were not pneumatised. In 49 sides (47.1%) the vidian canal was on the same plane as that of medial pterygoid plate in the coronal section (Fig. 1) In most right sides, the vidian canals were on the same plane as that of medial pterygoid plate. However, on the left sides, a greater number of vidian canals were found lateral to medial pterygoid plate. In those instances where vidian canal was not on the same plane as that medial pterygoid plate, the distance from this plane was measured. vidian canals were found at an average of 2.09 ± 1.04 mm lateral or 1.99 ± 1.01 mm medial to the medial pterygoid plate.

**Figure 2:**
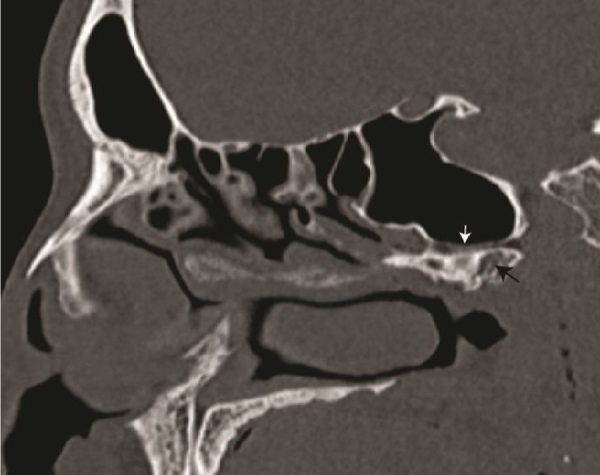
Sagittal computed tomography scan slide showing the vidian canal (white arrow) and a pneumatised pterygoid process (black arrow).

**Figure 3:**
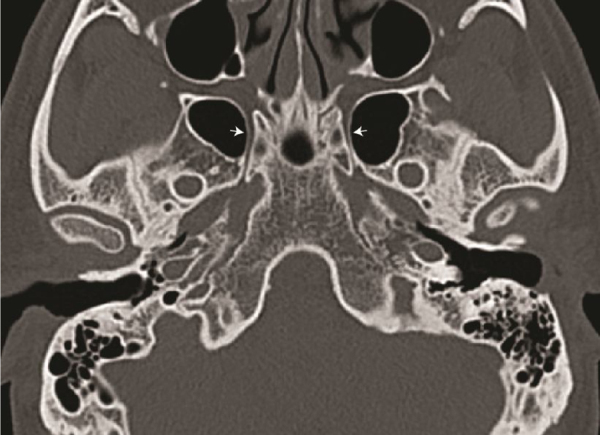
Axial computed tomography scan slide showing the vidian canal (white arrow) for length measurement.

Vidian canal partially protruded into the sphenoid sinus (type 2) was the most common type found both on right and left sides (Fig. 2 showing the same in sagittal section). Type 2 was found in 50.9% of the sides studied. The mean length of the vidian canal was 16.16 ± 1.8 mm (Fig. 3). There was no statistical difference between right and left sides in the length of vidian canal (Student *t*-test, 0.37). The relationship of the anterior end of vidian canal to the internal carotid artery (petrous part) in the coronal plane was inferolateral in 96 sides. The vidian canal was inferior to the internal carotid artery in 8 sides.

With 2 × 3 contingency table (Table 2), the significance of association of pterygoid process pneumatization was evaluated by Fisher Exact probability test. PA value was 0.000015 and PB was 0.000015. Chi-Square Test (df = 2) value was 20.8. Overall, p-value was 0.00003. This indicates statistically significant association between pterygoid process pneumatization and type of vidian canal. Cramer's V value from this table was 0.4472, indicating very strong association among the levels of the row and column variables.

**Table 1:** Tabulation of parameters noted in relation to vidian canal, pneumatization of sphenoid bone and medial pterygoid plate.

Parameter	Right side	Left side
Pterygoid process pneumatised		
Yes	27	30
No	22	25
Relation of vidian canal to medial pterygoid plate		
Medial	13	9
Lateral	10	24
Same plane	29	20
Type of vidian canal		
Type 1	20	16
Type 2	24	29
Type 3	8	7
Length of vidian canal	16.32 ± 1.68 mm	16 ± 1.97 mm
Relation of VC with ICA		
Inferolateral	48	48
Inferior	4	4

**Table 2:** A 2 × 3 contingency table showing association of pterygoid process pneumatization and type of vidian canal.

Pterygoid process pneumatization	Type 1	Type 2	Type 3	Total
Yes	10	33	14	57
No	26	20	1	47
Total	36	53	15	104

## Discussion

The vidian canal, being a deep-seated structure in the skull base, exacting delineation of its radiological anatomy will facilitate better understanding of pterygopalatine fossa structures. Defining the vidian canal in relation to pneumatization of pterygoid process and in relation to medial pterygoid plate will facilitate the identification of the canal.

In our study, the most common vidian canal configuration was type 2. This is in accordance with the Lee at al. study where they have reported 47% incidence.^14^ Similarly, Yazar et al.^9^ and Mohebbi et al.^20^ noted type 2 as most common configuration, with 54% incidence. In addition, they have reported cases with bony septum within the vidian canal. This probably divides the canal into two parts, for nerve and vessels. Contradicting these reports, Liu et al. have reported more type 1 vidian canal configuration.^21^

According to Yegin et al., the incidence of type 1 and type 3 are of almost equal incidence.^2^ Kazkayasi et al. reported types of vidian canals, notes bilateral protrusion only in half of type 2 configuration.^15^

Yegin et al. in addition to vidian canal types have also studied the different configurations of the sphenoid sinus floor.^2^ They report almost equal incidence of type 1 and type 3 varieties. In addition, they also note a dehiscent vidian canal in nearly one fourth of all scans studied. There is wide disparity in reports on vidian canal types. Kazhayasi et al. have also noted that pterygoid process pneumatization influences the degree of protrusion of the vidian canal.^15^ In their report, they have noted wide variation in types of vidian canal within individual sides.

The mean length of the vidian canal reported varies from 10 to 18 mm.^9^, ^14^, ^19^, ^20^^,^^22^, ^23^ The average length of the vidian canal from this study was 16.16 mm, which is within the reported range of previous studies. In addition, Adin reports sexually dimorphic vidian canal lengths, with slightly more length in males.^18^ However, we have not found such a significant difference between males and females. Also, there was no difference between right and left side.

Of particular note, among limited studies reported from India, Kirtane et al. have evaluated the use of vidian neurectomy for crocodile tears, a sequelae lesion of the facial nerve.^24^ Bhanu Pratap Singh et al.,^25^ Sushil Kumar Kashyap et al.^26^ and Prabu SS^27^ have noted protruded and dehiscent vidian canal while studying variations in sphenoid sinus. They have not evaluated the degree of pterygoid process pneumatization. Thakar et al. have evaluated the enlargement and dehiscence of the vidian canal among 16 patients with juvenile angiofibroma.^28^ They conclude that vidian canal involvement in angiofibroma is universal with almost all cases, harbouring microscopic residual tumor even after complete excision. Manna Joseph et al. have dissected ten cadavers for evaluating the relationship of internal carotid artery and vidian canal.^29^ They conclude that the carotid artery is superior and medial to vidian canal, thus drilling inferior and medial to vidian nerve provides the safe access to the anterior genu of internal carotid artery.

In this study, the association between pterygoid process pneumatization and type of vidian canal was evaluated with Fisher exact probability test. It was found to be statistically significant association between the pterygoid process pneumatization and type of vidian canal. The pneumatization process, if it extensively involves the sphenoid bone, there is more probability of the vidian canal gets either protruded into the sinus with a stalk (type 3) or will be present at the floor of sphenoid sinus (type 2).This study helps to predict the type of vidian canal after noting the pneumatization of pterygoid process.

Intra-operative identification of the vidian canal is of paramount significance while performing functional endoscopic sphenoid sinus surgeries. The close relation of the internal carotid artery makes the vidian canal an important landmark for identification of petrous and clival part junction of internal carotid artery. This study delineates the relative positions of types of vidian canal in relation to pneumatization of pterygoid process. As noted in many previous studies, the vidian canal was inferior to the junction of the petrous part and Gasserian (ascending) part of internal carotid artery.^7^, ^8^, ^19^, ^29^ We did not find any medial location of vidian canal ending at the petrous part of internal carotid artery.

## Conclusion

There is a statistically significant association between pterygoid process pneumatization and occurrence of type 2 and type 3 vidian canal configuration. The average length of the vidian canal was 16.16 ± 1.8 mm. Type 2 was found in 50.9% of the sides studied. In 49 sides (47.1%) the vidian canal was on the same plane as that of medial pterygoid plate. In most cases, the anterior end of vidian canal was inferolateral to the petrous part of internal carotid artery in the coronal plane.

## Conflicts of interest

The authors declare no conflicts of interest.
